# Impacts of risk and competition on the profitability of banks: Empirical evidence from Pakistan

**DOI:** 10.1371/journal.pone.0224378

**Published:** 2019-11-11

**Authors:** Faluk Shair, Na Sun, Sun Shaorong, Firdos Atta, Muhammad Hussain

**Affiliations:** 1 Business School, University of Shanghai for Science and Technology, Shanghai, China; 2 Lasbela University of Agriculture, Water and Marine Sciences, Uthal, Pakistan; The Bucharest University of Economic Studies, ROMANIA

## Abstract

The purpose of this paper is to investigate the impact of risk and competition on the profitability of the Pakistani banking industry. Data are retrieved from the annual statements of banks, the Ministry of finance Pakistan and the World Bank covering the period of (2007–2017). Two steps Generalized Method of Moments (GMM) with the collapse command is used as an estimation technique to overcome endogeneity, unobserved heterogeneity and autocorrelation problems. The results of the study showed that the liquidity risk has positive while credit risk, insolvency risk and competition hurt negatively the profitability of Pakistani banks. The results of the study also revealed that capitalization, size, taxation and GDP growth rate positively affect the Banks’ profits while banking sector development and infrastructure negatively affect banking profitability in Pakistan. The operational cost management positively affects net interest margins but negatively affects ROA and PBT in the Pakistani banking industry.

## Introduction

Financial institutions are considered as an engine of economic growth for developing countries because of their contribution to overall GDP. They play an intermediate role between lenders and borrowers, which help in economic growth. So, the better performance of banks not only ensures financial stability but also contributes to the economic growth of a country. Profitability is an important indicator of the banks’ performance which is widely used in the empirical literature [1–8]. However, profitability is a complicated issue because higher profitability may raise concerns about the potential abuse of market power and risk-taking behaviors of banks[9]. Several studies that focused on the relationship between competition and profitability in banks test the structure-conduct-performance (SCP) and efficient-structure (ES) hypotheses. The earlier hypothesis suggests that in markets with a lower level of competition few large banks can set higher prices to obtain more profit. The researchers in favor of the structure-conduct-performance hypothesis have found a significant impact of market power on the profitability of banks [10–13]. On the other hand, the second group of scholars supports the efficient-structure hypothesis as they have found no relationship between market power and profitability. They believe that it is the efficiency rather than market power that helps to increase or decrease the profitability of banks[14, 15].

The influence of competition on the profitability of banks may call for policy interventions because if the higher profitability comes from market power then it could negatively affect the customer in the form of low deposit rates, higher loan rates and poor quality of financial services. On the other hand, lower profitability may also result from technical inefficiency or extensive competition among banks, either of which requires a suitable policy response from policymakers for balancing competition and enhancing the managerial skill. The risk is another important factor that can affect profitability in either way. There are numerous studies available that focused on the risk and profitability of banks [7, 16–22].

In Pakistan, Financial assets to GDP ratio was 74.7% in 2017 out of which 55.3% is contributed by banks so the performance of the banking sector in Pakistan cannot be overlooked[23]. The remainder is contributed by Central directorate of national saving (CDNS) 10.6%, Insurance companies 4%, Non-banking financial institutions 3.5%, Development financial institutions 0.7% and Microfinance banks 0.7%, This study is conducted to examine the impact of risk and competition on the profitability of the Pakistani banks. The Lerner index and the Boone indicator are used to measuring competition in the Pakistani banking industry. We estimate the regressions based on the two-step GMM model to control the endogeneity problem among risk, competition and profitability. This study extends the existing literature of [17] in several ways. It includes the liquidity risk that has become the top priority of management to ensure the availability of funds for future demands at reasonable costs[24]. Another variable ‘infrastructure development’ is also used in this study. This variable is measured by the number of mobile subscribers per 100 peoples in the country to capture the impact of mobile subscribers on the profitability of banks in Pakistan. Another major difference of this study is its application to the Pakistani banking market which is still a fertile area to be examined. In Pakistan the private, foreign, state-owned and Islamic banks work together, so its results could add to the existing literature in a more meaningful way. Several other reasons make the Pakistani banking industry interesting to study.

First, the Pakistani banking industry has undergone dramatic changes since 1990 after several rounds of reforms. Three recent major reforms named poverty registration, getting credit and trading across borders created an efficient business environment for the Pakistani banking industry.

Second, the significance of a sound financial system has emerged after the introduction of the China-Pakistan Economic Corridor (CPEC) because of Pakistani banking industry likely to have a significant amount of share in CPEC projects. CPEC is expected to bring many opportunities for foreign and local investors to extend their business activities that may lead to enhance the demand for banks’ loans and influence the profitability of the Pakistani banking industry.

Third, geographically the position of Pakistan is considered as a strategic one, so the phenomenon of globalization and regional connectivity also increased the importance of Pakistani banking industry around the globe[25].

Finally, the political and economic conditions of developing economies are quite different from developed countries; therefore, the banking theories developed in advanced countries may not be the same in emerging economies like Pakistan.

The rest of the paper is structured as; in the next section, the history of reforms and development in the Pakistani banking industry is elaborated. Section 3 contains the existing literature about banks’ competition, risks and profitability. Variables description and the empirical model used in this study are given in Section 4. Research methodology and empirical results of the proposed model are given in sections 5 and 6 respectively. Finally, the article ends up with a conclusion in section 7.

## History of reforms and development in the Pakistani banking sector

Financial developments usually foster economic growth in developing countries and the financial institutions are considered an engine of economic growth. Financial developments are also playing a vital role in the economic growth of Pakistan [26]. Financial assets to GDP ratio in Pakistan remained 74.7% in 2017 out of which 55.3% is contributed by banks, so the performance of the banking sector in Pakistan cannot be overlooked [23]. However, such contribution of banks does not come out of sudden; the banking industry in Pakistan witnessed several developments and reforms. In 1960-70s, during the nationalization period, the government took control of all banks by imposing several restrictions on banking activities with a major focus on the credit ceiling. These restrictions resulted in nonproductive lending activities because of political interference rather than project viability. To overcome these problems other banking sector reforms initiated and implemented in the early 1990s. The government decided to privatize state-owned banks and liberal entry of new banks through these reforms. It triggered the competition in the banking sector which further improved internal efficiency and mitigated the cost of lending to ensure the access of finance to the middle class [27]. The State Bank of Pakistan [23] further removed business restrictions in the banking sectors by changing the interest rate structures, eliminating concessional lending schemes and lifting the cap for project financing in 1997–98 [28]. These reforms not only assert a positive influence on society by mobilizing the resources to productive sectors at competitive prices but also strengthen the performance of banks.

In 2017, 33 banks were operating in Pakistan, together with private (20), state-owned (5), foreign (4) and specialized banks (4). The assets, liabilities and owner equity of the Pakistani banking sector increased by 15.91%, 17.16% and 2.89% respectively in 2017 as compared to the previous year. The ROA declined from 1.21% to 0.84% and ROE from 16.06% to 12.41% in 2017 as compared to 2016. Sound and strict policies of the state bank of Pakistan (SBP) helped to reduce non-performing loans to gross advances ratio from 9.23% to 8.14% during the same period.

In a nutshell, several reforms helped to strengthen the Pakistani banking sector by increasing competitive conditions and mitigating risk-taking behavior. Nevertheless, as far as my knowledge is concerned, the impacts of these two factors (competition and risk) on the profitability of Pakistani banks have never been studied before. This article focused on these factors while taking into account the profitability of the Pakistani banking industry.

## Literature review

### Market power in the banking industry

Up till now, two approaches, structural and non-structural are commenced to measure market power in the banking industry across the globe. The structural approach is based on the structural-conduct-performance (SCP) hypothesis and the efficient-structure hypothesis. The former paradigm was initially developed by [29, 30] while the efficient structure (ES) hypothesis was introduced by [31]. SCP is measured by concentration ratios and argues that market structure significantly affects the competitive behaviors of banks in highly concentrated markets. In such highly concentrated markets, a small unit of large banks can set higher prices to earn more profit, so performance is derived from market structure. On the contrary, the efficient structure hypothesis claims that banks increase their market share by their efficient performance. This extension of size and market share further helps banks to increase their profitability. Concentration ratios and HHI (Herfindahl–Hirschman index) are commonly used methods to measure competition mostly in developing countries because of their simplicity and less data requirement. The concentration ratio method usually uses C3 and C5 ratios (share of large three and five banks) whereas; HHI focuses on the sum of the square of the market share of all banks operating in their respective markets. However, these methods use market share and market structure as proxies of competition which is considered as their major weakness along with the probability of endogeneity problem.

To overcome these flaws new empirical industrial organization (NEIO) introduced some new techniques for the direct measure of competition by observing the performance of firms in the market. These non-structural approaches include the conjectural variable model, Panzar-Rosse H-statistics, Lerner index and Boone indicator. The conjectural variable model introduced by [32] and [33] and is measured by the mark-up of price to marginal cost. Its value ranges between zero to one, where zero value represents that mark-up price and marginal costs are equal which means the industry is operating perfect competition. If its value is equal to 1, it indicates that there exists a monopoly in the market. The number of scholars used this model to measure competition in the banking industry around the globe[34, 35].

The H-statistics introduced by [36] vindicated that banks behave differently according to their market structure. On the other hand, [37, 38] suggested that in addition to the market structure the behavior of the banks also affected by the barriers for entry and exit, various activity restrictions and foreign ownership in any banking market. H-statistic applied by many scholars in advanced countries to measure competition in the banking industry [39, 40, 38, 41–44].

The Lerner index developed by [45] is consistently used in recent empirical studies as it is a direct measure of competition. Because of its ability to measure competition for an individual bank in each year, it is usually preferred over H-statistics which provides an aggregate measure for competition. It is the only method that is capable of measuring competition annually for each bank. The Lerner index remained the priority of many scholars in recent studies to measure competition among banks [46–51, 17].

The Boone indicator was introduced by [52], it is consistent with the efficient-structure hypothesis and argues that efficiency leads to increase market share which further increases profitability. The negative value of the Boone indicator indicates higher competition while a positive value identifies a lower level of competition. Several researchers used this approach to measure competition in the banking industry [53–55].

This study uses the Lerner index and the Boone indicator approaches to measure competition in the Pakistani banking industry. We use the Lerner index as it is a flexible indicator that does not require defining the relevant market and can be calculated with a limited number of observations. The Lerner index can measure market power for every bank in every year, so it helps to study the evolution of bank pricing behavior over time. However, this method has been criticized for not being able to fully capture competition as it is to measure the pricing of market power. To overcome this weakness we also used the Boone indicator which is considered a more suitable tool for measuring competition, particularly in developing countries. This method does not require information about prices when the costs of the banks are assessed by average costs. However, the monotonic and continuous relationship between profits and costs of banks is a major advantage of the Boone indicator.

### Risk and profitability in banks

Many kinds of risks naturally emerge from various banking operations. Literature shows us that scholars used different kinds of risks in their studies depending on their research objectives. This study uses the credit risk, liquidity risk and insolvency risk to observe their impact on the profitability of Pakistani banks. Lending is a major source of banks’ profitability but the credit risk arises when borrowers cannot repay their borrowed amount because of their financial problems. Many scholars measured credit risk by the ratio of loan loss provisions to total loans and observe its impact on banks’ profitability. However, those scholars came up with different findings like [2, 56, 57] found its negative impact on the profitability, while [17, 21] argued that it did not had any effect on the profitability of Chinese banks.

Liquidity risk is another important type of risk for banks because when the banks face liquidity problems they need to borrow extra money immediately with extra cost to meet their cash needs for day to day operations. Liquidity risk could not only hurt reputation but can also lead to insolvency of banks[58]. Some studies measure the liquidity risk by the ratio of liquid assets to total assets, where a higher ratio indicates lower liquidity risk [59–64]. Several researchers found a negative impact of liquidity risk on the profitability of banks by arguing that holding of most liquid assets results in lower returns [65–67].

Z-score is used as an inverse proxy of insolvency risk for banks in many studies [68–72]. It is measured as the sum of banks’ return on assets and equity to the ratio of total assets over the standard deviation of banks’ return on assets.

### The determinants of banks’ profitability

The profitability of the banking sector has remained the center of focus for academicians in developing as well as developed countries and has been widely investigated theoretically and empirically. Some studies were also conducted in Pakistan to find out the important factors of banks’ profitability and came up with different findings, like [73] argued that banks’ profit is positively associated with proper management of assets and GDP of the country and negatively related to the capital adequacy, credit risk, operating efficiency and consumer price inflation rate. Similarly, [74] used four profitability indicators (Return on assets, Return on equity, Return on capital employed and Net interest margin) and by using Ordinary Least Square method revealed that Return on assets of the Pakistani banks increased with the increase of banks’ size, loans, deposits, GDP and inflation. Return on equity was also positively influenced by size, deposits, GDP and inflation while negatively affected by banks’ capital and market capitalization. Return on capital employed was positively affected by size while negatively influenced by capital, loans and market capitalization. The Net interest margin increased with loans and inflation while decreased with GDP and banks’ capital. Finally, [25] studied to find out the internal determinants of banks’ profitability in Pakistan. The study used the Fixed-effect model with one year lag of all explanatory variables to mitigate the potential problem of endogeneity. The results showed that bank size, credit risk and financial stability have a positive relationship with banks’ profitability while the funding risk and financial crises have a negative impact on banks’ profits.

Some of the other studies were conducted to define the key characters of banks’ profitability in individual countries while some focused on a panel of countries. The second group mostly kept a focus on the European banking industry [75, 76, 56, 77, 57]. Some of the empirical studies which focused to find determinants of profitability in individual countries, including UK [78–81], Australia [82], China [7], Turkey[83], Switzerland [84], Philippines [85].

Empirical findings of all the above studies vary because of the disparity in data, period, and their applications to different countries. Besides, the above differences, we deem it would be better to classify the determinants of bank’s profitability in two categories. In the first category, we place all determinants which are under the control of management and considered as internal factors. The second category contains external factors which are beyond the control of management. These determinants are selected according to the nature and purpose of the study. Internal factors are termed as bank-specific variables which include bank size, operational cost management, capitalization, and various risks. External factors are macroeconomic and industry-specific, including banking sector development, infrastructure development and economic growth.

In previous literature, we observed the banks’ profitability is a function of the external and internal factors. Somehow the internal factors are relatively more effective towards profitability. Most of the prior studies have a deep insight into those factors and stated that size [86, 87], capital ratios [67], operational efficiency and asset quality are the most important determinants of banks’ profitability.

This article has filled up a gap in the literature by analyzing the impact of competition and risk together with the bank-specific, industry-specific and macroeconomic factors on the profitability of the Pakistani banking industry.

## Model and variables

The impact of risk and market power on the profitability of Pakistani banks is estimated by following the model proposed by [88] and [17], expressed as:

$${\text{Profit}}_{\text{it}}=\text{C}+{\delta \text{Profit}}_{\text{i},\text{t}-1}+\sum_{\text{j=1}}^{\text{j}}\beta_{\text{j}}{\text{X}}^{\text{j}}{\;}_{\text{it}}+\sum_{\text{l}=1}^{\text{l}}\beta_{\text{l}}{\text{X}}^{\text{l}}{}_{\text{it}}+\sum_{\text{m}=1}^{\text{m}}\beta_{\text{m}}{\text{X}}^{\text{m}}{}_{\text{it}}+\;\Upsilon Z_{\text{it}}+{\text{e}}_{\text{it}}+{µ}_{\text{it}}$$ (1)

Where the subscript i refer to the individual bank and t refers to the time (year); and δ, β, γ are estimated parameters where δ represents the speed of adjustment to equilibrium. The value of δ ranges between zero to one, where the higher value represents the less competitive structure and the slower adjustment while the lower value of δ shows the faster adjustment to the equilibrium and stronger competition. Profit_it_ represents the profitability indicators of particular bank i in specific year t and is measured with PBTTA, ROA_,_ ROE and NIM. X_it_ represents determinants of bank’s profitability which are further categorized into three groups, bank-specific determinants X^j^_it;_ industry-specific determinants X^l^_it_ and macroeconomic determinants X^m^_it_. Z_it_ represents the group of four dummy variables used in the study; one is financial crises which are used to capture the impact of financial crises (2008–2009) on the banks ‘profitability. The other three dummy variables are the State-owned banks, the Islamic banks and the foreign banks which are used in the model to observe their impact on the profitability of the Pakistani commercial banks.

### Dependent variables

The objective of the paper is to analyze the impact of risk and competition on the profitability of Pakistani banking industry while controlling bank-specific, industry-specific and macroeconomic variables. Four profitability indicators net interest margin (NIM), return on assets (ROA), return on equity (ROE) and the ratio of profit before tax to total assets (PBT) are considered as dependent variables in this study.

#### Profitability before tax to total assets (PBT)

**Fig 1:**
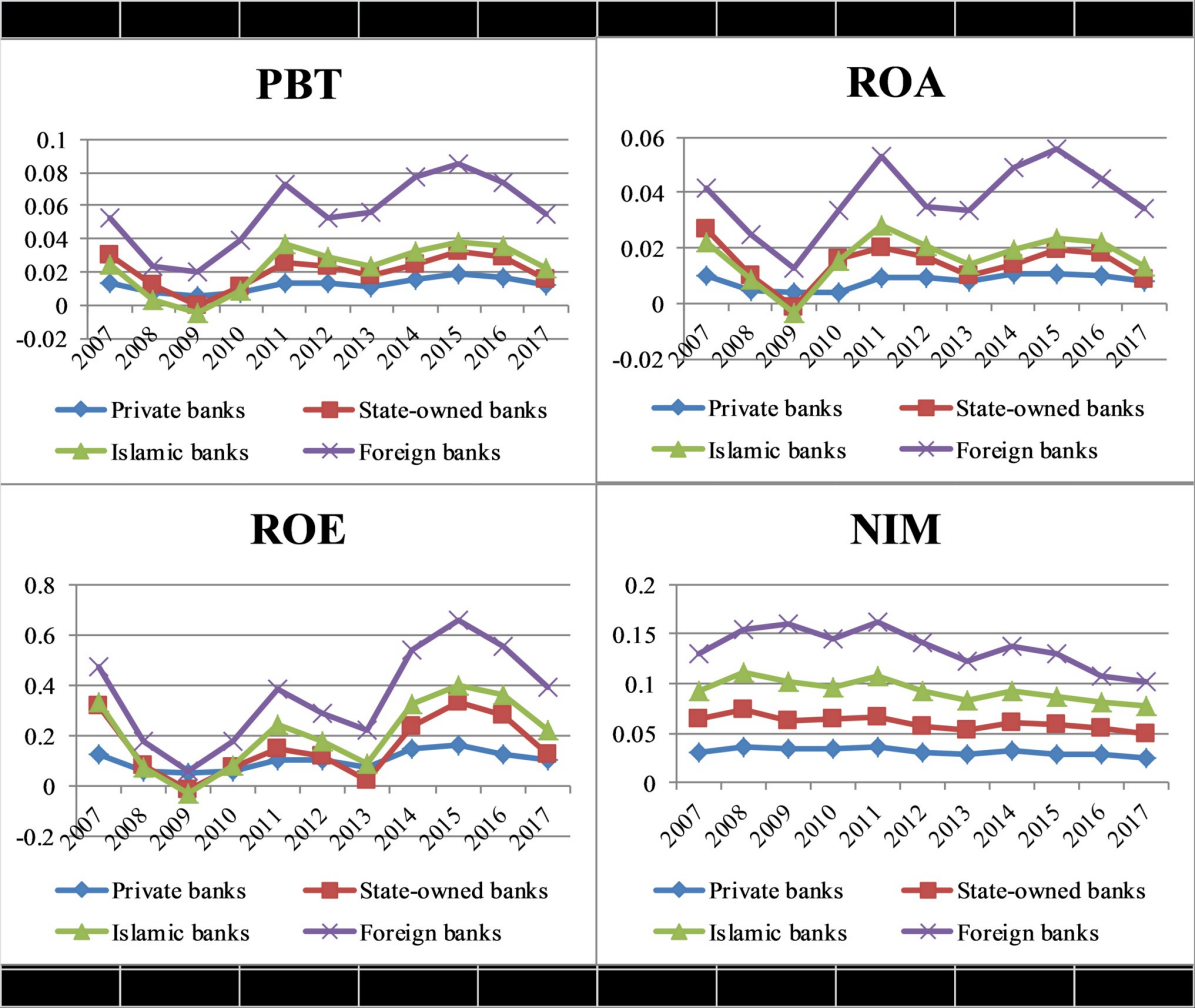
Profitability indicators of Pakistani banks.

Here, profitability margin is used as a measure of profitability; it differentiates from ROA as it measures the profitability by excluding taxes. The reason for including this indicator is to test out the influence of taxes on the profitability of Pakistani banks. When we measured the profitability through profit margins, it showed that the foreign banks have relatively high profit-margins than that of the state-owned, private and Islamic banks as shown in Fig 1.

#### Return on assets (ROA)

This profitability ratio shows banks' ability to earn profits through their total assets engaged in the business. It is considered a key ratio for the measurement of profitability and widely used in the empirical literature [89–91, 17]. This ratio helps to see the ability of the banks’ administration to utilize its investment and financial resources for earning profits [92]. While measuring the ROA of Pakistani banks, Fig 1 demonstrates the foreign banks utilized their financial resources in a more meaningful way as compared to other banks.

#### Return on equity (ROE)

It represents the profitability of a bank which is generated through the invested amount of its shareholders.[84] Claimed that it is not a good measure of profitability as it does not focus on leverage risk. They support their claim by arguing that banks usually with a higher level of equity (low leverage) have higher ROA but lower ROE. However, this indicator is important to study because it reveals how well bank management is in using shareholder's funds. ROE helps to measure the efficiency of a bank for utilizing investment funds to cause earning growth.

Fig 1 suggested that ROE of the state-owned banks and private banks showed more fluctuation as compared to the foreign and Islamic banks in Pakistan.

#### Net interest margin (NIM)

This accounting-based measure is widely used in empirical studies as a profitability indicator [76, 84, 17, 19]. It represents the accounting gauge of interest revenue as a share of its interest-bearing assets during a specific time. It identifies the earning capacity of banks through the utilization of all assets and also the ability of banks to make the right decisions regarding banking spreads relative to its interest expenses. The foreign Banks in Pakistan showed better performance through this indicator as shown in Fig 1

### Independent variables

Explanatory variables can be categorized into banks’ specific, industry-specific and macroeconomic determinants. Bank specific-determinants include size, capitalization, taxation, operational cost management, credit risk (LLPTL), insolvency risk (Z- score) and liquidity risk. The macroeconomic determinants comprised of GDP growth rate and infrastructure development, while Industry-specific variables include banking competition (measured by the Lerner index and the Boone indicator) and banking sector development.

### Bank specific variables

#### Size

Natural logarithm of total assets is taken as a proxy of banks’ size which frequently used in empirical literature as [88, 84, 17]. However, there exists a contradiction among scholars about the behavior of size towards profitability. Some of them viewed that larger banks usually reduce their cost by getting the edge from economies of scale and scope which ultimately boost profitability [93–97, 67]. Conversely, the problem of information asymmetry decreases along with size, so this problem is reduced by specialized and smaller banks, proceeding the negative impact of size towards profitability[98]. Smaller banks can get economies of scale up to a certain limit beyond which further size enlargement will result in diseconomies of scale [99]. The above argument is sustained by [88], by suggesting that banks’ profit move along with size to a certain limit then starts to decline because of bureaucratic and other reasons. These contradictory opinions leave the door open for us to expect its impact on profitability in either way.

#### Capitalization

The ratio of shareholders’ equity to total assets is considered as a proxy of capitalization, which is followed by [88, 84, 17]. Some studies proposed a negative relationship between capitalization and banks’ profitability by arguing that a higher level of capitalization results in lower risk position in banks which leads to lower returns [100, 84]. Conversely, the banks with high capital are in a better position to absorb risk, so they engaged in more lending activities which lead to an increase in their profitability in the form of interest revenue. Another important point to support this argument is the banks with high capital ratios are capable of mitigating their funding cost as they have more creditworthiness. Based on the above discussion, we do not have any prior expectations regarding the impact of capitalization on the profitability of Pakistani banks.

#### Taxation

The ratio of tax to operating profit before tax at the end of each year in the sampled period is used as a measure of taxation. Higher tax payment increases the cost of banks which can ultimately affect negatively to the profitability. Hence, we expect the negative impact of taxation on the profitability of banks which is also consistent with the findings of [19].

#### Operational cost management (MOC)

The motivation behind the selection of this variable is its extensive use in prior studies like [84, 90, 101, 17].Efficient banks are capable of reducing their operating cost successfully which ultimately increase their profitability [88, 102, 103].On the other hand, [67, 104] argued that higher operational cost is linked with salaries and wages of employees, while higher wages and salaries paid to staff significantly improve labor productivity which ultimately brings improvement in profitability. So there is no prior expectation about the relationship between operational cost management and banks’ profitability.

#### Risk

The credit risk is measured by the ratio of loan loss provision to total loans (LLPTL).The coefficient of this factor is expected to be negative as bad loans negatively affect the profitability of banks. Greater exposure of banks towards high-risk loans led to more non-performing loans which ultimately negatively affect the profitability of banks [105].

Z-index is used as an insolvency risk which is widely used as a risk/stability indicator in the empirical literature [72]. This risk determinant is measured as the sum of banks’ return on assets and equity to the ratio of total assets over the standard deviation of banks’ return on assets. Z-index is an inverse proxy of banks’ insolvency risk, so we assume a positive relationship between the Z-score and profitability of banks.

The ratio of liquid assets to total assets is taken as liquidity risk which is widely used in previous studies [62–64]. The lower value of this ratio means more liquidity risk. Following the studies of [65–67] we expect a negative relationship between this ratio and profitability because holding more liquid assets can result in less profitability.

### Industry-specific determinants

#### Competition

The Lerner index and the Boone indicator are used to measuring banks’ competition in this study. Studies in favor of SCP hypothesis suggested that less competition in banks tend to expand their business activities and higher profitability. Conversely, the competition-efficiency hypothesis recommended that efficiency leads to reduce banks’ costs which further proceed to higher profitability. We do not have any prior expectation about the impact of this variable on the profitability of Pakistani banks.

#### Banking sector development

The ratio of overall assets of the banking sector to GDP is used as a proxy of this determinant. By following the recommendations [19], we expect a positive impact of this determinant on the profitability of banks.

### Macroeconomic variables

#### GDP growth rate

Some studies suggested a positive influence of GDP growth rate on the profitability of banks [88, 76]. These studies favor the argument that during the boom period of an economy the lending activities of banks expand which further leads to increase profitability. On the contrary side, [19] found a negative relationship between GDP growth rate and banks. So we keep this scenario open for our study.

### Infrastructure development

We used Infrastructure development as a macroeconomic indicator that is measured by the number of mobile subscribers per 100 persons in Pakistan during the study period. The higher value of this indicator means a country has better IT/telecommunication infrastructure. The resulting advancement in mobile banking will reduce the cost which further leads to an improvement in the profitability of Pakistani banks. So, we expect a positive relationship between infrastructure development and banks’ profitability.

Table 1 represents the measurement of all variables, sources for data collection and the expected impact of explanatory variables on the profitability of banks.

**Table 1:** Measurement of variables and their expected impact on the banks’ profitability.

Dependent Variables	Measurement of variables	Source of data collection	Expected impact
PBTTA	Banks profitability before taxes/Total assets	Bank scope	
ROA	Net profit after tax/Total assets	Bank scope	
ROE	Net profit after tax/Total shareholders’ equity	Bank scope	
NIM	(Total Interest income-Total interest expenses)/(Total assets)	Bank scope	
** Independent variables**			
*** Banks’ specific variable****s*			
Size	Natural logarithm of total assets	Bank scope	?
Capitalization	Total shareholder equity/Total assets	Bank scope	?
Taxation	Tax/ Operating Profit before tax payment	Bank scope	Negative
Operational cost management	Operational cost/Total assets	Bank scope	?
Insolvency risk(Z-score)	(see the portion for measurement of the risk)	Bank scope	Positive
Credit risk	Loan loss provisions/Gross loans	Bank scope	Negative
Liquidity risk	Liquid assets/total assets	Bank scope	Negative
***Industry-specific variables***			
The Lerner index (competition measure)	(see portion measurement of the Lerner index)	Bank scope	?
The Boone indicator(competition measure)	(see portion measurement of the Boone indicator)	Bank scope	?
Banking sector development	Total banking assets/GDP	Finance ministry of Pakistan& Bank scope	Positive
***Macroeconomic variables***			
GDP growth	The annual GDP growth rate	The finance ministry of Pakistan	?
Infrastructure development	No of mobile subscribers per 100 persons	World bank	Positive

### Measurement of risk and competition

#### Measurement of insolvency risk

The study uses Z-index as an inverse proxy of banks’ insolvency risk, which combines profitability, leverage and return in volatility in a single measure. It is widely used as a risk/stability indicator in the empirical literature [47, 51, 17].

Z-index is given by the ratio:

$$Z-Index_{it}=\frac{ROA_{it}+\frac{E_{it}}{TA_{it}}}{\delta ROA_{it}}$$ (2)

Where

ROA_it_ = Average return on assets of a bank i in period t.

E_it_/ TA_it_ = Average equity to total assets ratio of a bank i in period t.

*δ*ROA_it_ = Standard deviation in return on assets of each bank in a specific period.

A high value of the Z-index indicates the financial soundness and stability and low probability of credit risk. As it is an inverse proxy of credit risk, its lower value indicates a higher risk.

### Estimation of Banks’ competition

#### The Lerner index

Lerner index is developed by [45] and is extensively used to measure the market power of banks in recent studies, such as [47, 51, 106, 17, 107]. It is considered as a markup of price over marginal cost and deviation of price from marginal cost represents the market power of banks. The lower value of the Lerner index identifies more competition.

The Lerner index can be measured as:

$$Lerner\;index_{it}=\frac{PTA_{it}-MCTA_{it}}{PTA_{it}}$$ (3)

Where PTA_it_ refers to the price of total assets which is measured by dividing, total revenue (sum of total interest and non-interest income) to total assets for each bank i in at a specific period t following the studies of [108, 17] among others. Whereas the marginal cost of total assets for each bank i in period t is represented by MC_it,_ which is calculated by the translog cost function, following the methodology of [109, 94, 51].

$$lnTC_{it}=\delta +\gamma 1\left(lny_{it}\right)+\omega 1\left(lnw1_{it}\right)+\omega 2\left(lnw2_{it}\right)+\omega 3\left(lnw3_{it}\right)+\gamma 2{\left(lny_{it}\right)}^{2}+\gamma 3\left(lny_{it}\right)\left(lnw1_{it}\right)+\gamma 4\left(lny_{it}\right)\left(lnw2_{it}\right)+\gamma 5\left(lny_{it}\right)\left(lnw3_{it}\right)+\omega 4{\left(lnw1_{it}\right)}^{2}+\omega 5{\left(lnw2_{it}\right)}^{2}+\omega 6{\left(lnw3_{it}\right)}^{2}+\omega 7\left(lnw1_{it}\right)\left(lnw2_{it}\right)+\omega 8\left(lnw2_{it}\right)\left(lnw3_{it}\right)+\omega 9\left(lnw1_{it}\right)\left(lnw3_{it}\right)+e_{it}$$ (4)

In (Eq 4) i is representing a particular bank and t corresponds to a specific period. lnTC represents the natural logarithm of the total cost which is the summation of total interest, noninterest, administrative and other operating expenses. Y_it_ is representing output quality in the form of total assets. Three input prices w1_it_, w2_it_, w3_it_ are used to capture the price of the fund, price of labor and price of fixed capital respectively. To ensure symmetry and homogeneity in input prices, we impose the following restrictions:

ω1+ω2+ω3=1

γ3+γ4+γ5=0

ω4+ω7+ω8=0

ω5+ω7+ω9=0

ω6+ω8+ω9=0

Estimated coefficients of Eq 4) are used to compute marginal cost (MCTAit).

$$MCTA_{it}=\frac{TC_{it}}{Y_{it}}(\;\gamma 1+\gamma 2\text{ln}\left(Y_{it}\right)+\gamma 3\text{ln}\left(w1_{it}\right)+\gamma 4\text{ln}\left(w2_{it}\right)+\gamma 5ln\left(w3_{it}\right)$$ (5)

After computing the price of output and estimation of the marginal cost of total assets, we can measure the Lerner index for each bank in every year.

Table 2 shows a summary of all variables used for the estimation of the Lerner index.

**Table 2:** Variables used for estimation of the Lerner index.

Variable	Notation	Measurement
3Input prices	W1_it_	Price of funds = Interest expenses/Total deposits
	w2_it_	Price of labor = Personnel expenses/ Total assets
	w3_it_	Price of fixed capital = Administrative and other operating expenses/ Total assets
Output price	Y_it_	Total assets
Total cost	TC_it_	Sum of interest and non-interest expenses
Marginal cost	MCTA_it_	Estimated using Eqs (4) and (5).

#### The Boone indicator

In addition to the Lerner index we also employed the most recent approach the Boone index [110] to estimate competition in the Pakistani banking industry. Boone indicator also termed as profit elasticity which estimates the percentage loss when marginal cost is increased by one percent. This indicator supports the efficiency-structure hypothesis by arguing efficient firms perform better with the increase of competition. Hence the Boone indicator can be expressed by the following equation;

$$ROA_{it}=\alpha +\beta lnMC_{it}+e_{it}$$ (6)

**Fig 2:**
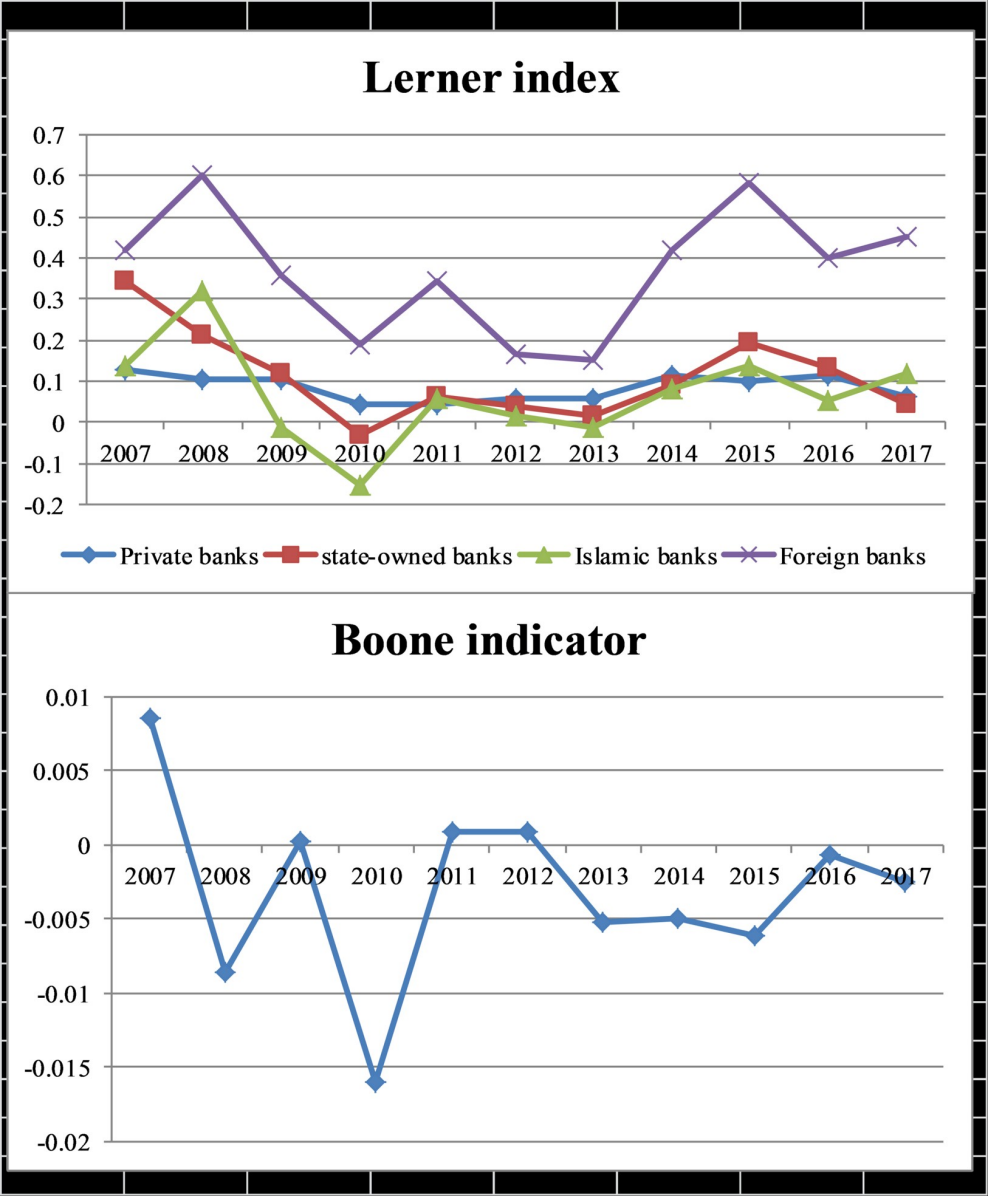
Competition indicators of Pakistani banks.

Where ROA_it_ refers to the profit of the bank i in time t and β is the profit elasticity also known as the Boone index. MC represents the marginal cost. Competition trend in Pakistani banks during the study period depicted in Fig 2.

### Methodology

#### Panel data

When sample data is comprised of time series and cross-sections, in that case, the use of panel data is considered as the most suitable tool. The most important feature of panel data is its ability to overcome constant, unobservable and heterogeneous characteristics of all banks included in the sample depending on the model selection. Several researchers used the fixed-effect model to observe the determinants of banks’ profitability [57, 85]. However, the fixed effect model cannot overcome endogeneity, unobserved heterogeneity and autocorrelation problems[17]. To fix those problems, we have selected the GMM model which is consistently used in recent studies [84, 111, 51, 17]. We used the system GMM model proposed by [112] and more precisely the two-step system GMM model of [113, 114] to get better estimates for the impact of risk and competition on the profitability of Pakistani banks. The two-step system GMM model is considered more appropriate for the conditions where the number of cross-sections exceeds to the number of years of sampled data, explanatory variables may correlate with the error term, and there exist heteroscedasticity and autocorrelation within individuals which are most common in banks level data. We used one-period lag of dependent variables (profitability indicators), we also used the collapsing method developed by [115] to limit the number of instruments because an important issue with the GMM model is ‘ too many instruments’ as the number of instruments exceed from the number of cross-sections[116]. The same method is also used by [117]. The one year lag of dependent variables, capitalization, liquidity risk, credit risk and Z-score are used as endogenous variables. We also tested the endogeneity of these variables with the two-stage least square method; all post- estimation results confirm the endogeneity problem of these variables. We tested all other variables through this method but found no endogeneity issue among other variables. In this study, we used STATA 15 for data analysis.

#### Sample

The sample data focus on the private, state-owned, Islamic and foreign banks working in Pakistan since 2007. We selected all 26 banks which were operating in Pakistan from 2007–2017. The values of all variables are in Pakistani rupees (in millions). The data is collected from the annual financial statements of banks, the ministry of finance Pakistan and World Bank for banks’ specific, macroeconomic and industry-specific variables respectively.

## Results

Table 3 reports the descriptive statistics for profitability indicators of Pakistani banks. The foreign banks and the private banks showed more volatility for earning PBTTA but they also enjoyed more PBTTA as compare to the state-owned and Islamic banks. Return on assets and net interest margin showed almost the same variations in private, foreign and state-owned banks. ROE displays wide variations for the state-owned banks (-23.1% to 37.3%). Thus ROE of Pakistani banks seems to be more volatile during the study period.

**Table 3:** Summary of profitability indicators.

Variables	Observations	Mean	Standard .Dev.	Min	Max
**Overall banks**					
PBTTA	286	.012	.017	-.024	.047
ROA	286	.008	.011	-.017	.032
ROE	286	.09	.135	-.231	.373
NIM	286	.032	.013	.001	.065
**Private banks**					
PBTTA	176	.012	.016	-.024	.047
ROA	176	.008	.011	-.015	.032
ROE	176	.102	.135	-.231	.365
NIM	176	.032	.013	.001	.065
**State owned banks**					
PBTTA	44	.008	.014	-.024	.037
ROA	44	.007	.01	-.015	.025
ROE	44	.055	.168	-.231	.373
NIM	44	.029	.014	.002	.061
**Islamic banks**					
PBTTA	44	.003	.012	-.024	.022
ROA	44	.002	.008	-.017	.015
ROE	44	.05	.1	-.162	.226
NIM	44	.033	.009	.009	.052
**Foreign banks**					
PBTTA	22	.033	.015	.001	.047
ROA	22	.021	.01	.001	.032
ROE	22	.149	.088	.01	.341
NIM	22	.043	.014	.017	.061

Table 4 identifies the descriptive summary of explanatory variables during the study period. The summary shows that during the study period the foreign and private banks showed more strength in terms of capitalization as compared to the state-owned and Islamic banks. The summary statistics revealed that during the study period higher taxes were paid by the foreign banks while the state-owned banks paid fewer taxes as compared to other banks. The ratio of operational cost to total assets (MOC) of the private and Islamic banks is higher than the state-owned and foreign banks which indicate that the state-owned and foreign banks managed their operational cost-efficiency. The higher values of the Z-scores indicates that the foreign and private banks are much stronger with having less probability of insolvency as compare to the state-owned and Islamic banks. The Islamic banks seem to be stronger by having the lowest ratio of loan loss provisions (4.1%) followed by the private (8.9%) and foreign banks (9.6%). The higher value of loan loss provisions in the state-owned banks also identifies their inefficiency in loan disbursement and loan collection.

**Table 4:** Summary statistics of explanatory variables.

Variables	Observations	Mean	Standard .Dev.	Min	Max
**Overall banks**					
Size	286	12.185	1.242	9.098	14.803
Capitalization	286	.103	.064	-.025	.349
Taxation	286	0.326	0.096	0.152	0.524
Operational cost management	286	.028	.01	.004	.06
Credit risk	286	.085	.055	.001	.232
Liquidity risk	286	.092	.036	.034	.186
Insolvency risk	286	117.738	186.501	-288.041	529.431
Banking sector development	286	.509	.036	.454	.58
GDP growth rate	286	.039	.014	.004	.058
Infrastructure development	286	62.874	9.891	39.204	73.357
**Private banks**					
Size	176	12.542	1.09	9.71	14.803
Capitalization	176	.1	.066	.003	.349
Taxation	176	0.327	0.097	0.152	0.524
Operational cost management	176	.027	.009	.004	.06
Credit risk	176	.089	.053	.003	.232
Liquidity risk	176	.081	.026	.034	.158
Insolvency risk	176	133.278	197.161	-288.041	529.431
Banking sector development	176	.509	.036	.454	.58
GDP growth rate	176	.039	.014	.004	.058
Infrastructure development	176	62.874	9.901	39.204	73.357
**State owned banks**					
Size	44	12.294	1.333	10.12	14.681
Capitalization	44	.083	.051	-.025	.187
Taxation	44	0.304	0.098	0.152	0.524
Operational cost management	44	.017	.007	.004	.036
Credit risk	44	.11	.047	.019	.201
Liquidity risk	44	.096	.036	.05	.171
Insolvency risk	44	85.197	171.362	-224.21	412.473
Banking sector development	44	.509	.036	.454	.58
GDP growth rate	44	.039	.014	.004	.058
Infrastructure development	44	62.874	9.987	39.204	73.357
**Islamic banks**					
Size	44	11.382	1.021	9.098	13.575
Capitalization	44	.102	.055	.047	.233
Taxation	44	0.327	0.093	0.152	0.524
Operational cost management	44	.034	.01	.005	.05
Credit risk	44	.041	.034	.001	.163
Liquidity risk	44	.115	.04	.056	.186
Insolvency risk	44	38.178	140.052	-262.43	495.977
Banking sector development	44	.509	.036	.454	.58
GDP growth rate	44	.039	.014	.004	.058
Infrastructure development	44	62.874	9.987	39.204	73.357
**Foreign banks**					
Size	22	10.719	.752	9.628	11.671
Capitalization	22	.161	.063	.069	.233
Taxation	22	0.367	0.077	0.152	0.524
Operational cost management	22	.038	.011	.017	.05
Credit risk	22	.096	.069	.003	.232
Liquidity risk	22	.132	.049	.055	.173
Insolvency risk	22	217.622	140.941	7.445	529.431
Banking sector development	22	.509	.036	.454	.58
GDP growth rate	22	.039	.015	.004	.058
Infrastructure development	22	62.874	10.106	39.204	73.357

The lower liquid assets to total assets ratio (liquidity risk) of the private banks show that they work aggressively and invest the extra amount to get more profit margins by accepting more liquidity risk. On the other hand, the foreign and Islamic banks keep more liquid assets with them and avoid paying extra borrowing costs when they need cash immediately. This shows the conservative behavior of the foreign and Islamic banks which can also reduce their profit margins.

Table 5 shows the annual position of all three input prices, the price of total assets, marginal cost, total cost, the Lerner index, and the Boone indicator from 2007–2017. The negative value of the Lerner index in 2015 indicates higher marginal costs and negative market power of the Pakistani banking industry. It shows the non-optimum behavior of the Pakistani banks in the aforementioned period.

**Table 5:** Annual summary statistics for variables of Lerner index and Boone indicator.

Year	W1	W2	W3	Pi	MC	Lerner	Boone indicator
2007	.0364322	.01245713	.02581143	.08373516	.07279063	.09977323	.00851792
2008	04646462	.01518557	.0332607	.10073147	.0883709	.11449802	-.00853367
2009	.05294363	.01391052	.030584	.40335343	.09889298	.06943276	.00020088
2010	.05153544	.01393136	.03022524	.0999831	.09482656	.01983561	-.01593579
2011	.05437221	.0134352	.02910839	.10302626	.09648965	.05075253	.0008781
2012	.04962281	.01692195	.02810829	.09422275	.08926454	.0408826	.00087407
2013	.04294531	01278527	.02698121	.08465388	.08003723	.03805849	-.00520213
2014	.04293807	.01223587	.02602082	.09208543	.08041801	.09162116	-.00498306
2015	03472991	.01161805	.02331524	.07920019	.06936003	-.05530439	-.00613646
2016	.0292789	.01109036	.02333594	.06968437	.06154025	.08814668	-.0006358
2017	.02882734	.010524	.02330783	.06706224	.0617442	.07317504	-.00255245

We examined the correlation coefficient of all explanatory variables to check the probability of multicollinearity among them. Table 6 identifies that there is a low probability of multicollinearity among variables which is unlikely to affect our econometric results because multicollinearity exists if the coefficient is greater than o.70[118].

**Table 6:** Correlation matrix.

	(1)											
	Lerner	Boone	Size	Capitalization	Taxation	OCM	CR	LR	IR	BD	GDP	ID
Lerner	1											
Boone indicator	0.0119	1										
Size	0.393^***^	0.0505	1									
Capitalization	-0.0708	0.0208	-0.578^***^	1								
Taxation	0.168^**^	0.133^*^	0.112	0.00139	1							
Operational cost management	-0.180^**^	-0.0708	-0.460^***^	0.436^***^	0.0369	1						
Credit risk	-0.194^**^	-0.00362	0.0584	0.0567	-0.148^*^	0.0563	1					
Liquidity risk	0.195^***^	0.0475	-0.198^***^	0.132^*^	-0.00766	0.198^***^	-0.263^***^	1				
Insolvency risk	0.426^***^	0.0470	0.0964	-0.144^*^	-0.00425	-0.207^***^	-0.294^***^	0.00485	1			
Banking sector development	0.0757	0.282^***^	0.0207	0.0428	0.0207	-0.134^*^	-0.203^***^	-0.0160	0.0114	1		
GDP growth rate	0.0564	0.386^***^	0.0989	0.0137	0.0526	-0.127^*^	-0.143^*^	-0.0852	0.0473	0.627^***^	1	
Infrastructure development	-0.0362	-0.141^*^	0.349^***^	-0.205^***^	0.112	-0.0874	0.165^**^	-0.299^***^	-0.0617	-0.229^***^	0.0418	1

^*^ *p* < 0.05,

^**^ *p* < 0.01,

^***^ *p* < 0.001, here OCM = operational cost management, CR = credit risk, LR = liquidity risk, IR = insolvency risk, BD = banking sector development, GDP = GDP growth rate and ID = infrastructure development.

Table 7 reports the empirical results for the impact of risk and competition on the profitability of Pakistani banks by using the Lerner index as a competition measure. Following the suggestion of [119] to consider the persistence of profitability using a dynamic panel model, we used lagged of dependent variables in our model. The significant coefficient values of lagged dependent variables (NIM.ROE, ROA and PBT) confirm the dynamic character of our model specification. The Hansen-j test is used to check the validity of instruments because of its consistency in the presence of heteroscedasticity and autocorrelation[120]. We used the system GMM model proposed by [112] for first and second order serial correlation. The results show autocorrelation exists in the first- order for all four models but it does not mean that our estimates are inconsistent. The inconsistency of the model would be implied if a second-order correlation exists [112], which is rejected by the AR(2) test. The significant positive value of the Lerner index with all four profitability indicators identifies that competition negatively affects the banks’ profitability in Pakistan, which is in line with the Structure- Conduct-Performance (SCP) hypothesis. As far as the impact of various types of risk is concerned, the significant positive value of Z-score with profitability (ROA and PBT) determines that lower insolvency risk boosted banks' profitability. The negative significant coefficient of credit risk for profitability (ROE) shows that credit risk is hammering negatively the return on equity of the Pakistani banks. The significant negative coefficient liquidity risk for (PBT) shows that the banks with more liquidity risk get more profits (PBT) because a lower amount of this ratio indicates higher liquidity risk. Turning to the other explanatory variables we found that the profitability of Pakistani banks (ROE, ROA, and PBT) increased with the bigger size. This result is consistent with the findings of [121, 122]. It may be due to the reduction of cost from economies of scale and better monitoring technologies of large banks to mitigate non-performing loans, which ultimately tends to enhance profitability. Financial crises negatively affected return on assets, return on equity and PBT while net interest margins of the Pakistani banks continued to increase during this period. The coefficients of capitalization show positive significant relationships with all profitability Indicators. It's because highly capitalized banks are usually less dependent on external funding which helps to reduce funding costs and increase their profitability. Besides this, highly capitalized banks are sound enough to endure their profitability even during economically challenging times. Contradictory to our expectation, the taxation showed a significant positive relationship with profitability (ROA), which means that the Pakistani banks successfully transferred their tax burden to the customers. Banks usually forward their interest burden to the customers by increasing the price of their financial services, so taxing banks can be interpreted as taxing financial services [123]. The impact of operational cost management on banks’ profitability is mixed, it positively affects net interest margins while negatively affect the return on assets and PBT of Pakistani banks. It shows when the banks decrease their operational cost; it can increase profitability (ROA, PBT) because the reduction of cost automatically increases profitability. On the other side, banks increase their operational cost by giving more compensation to their competent and hardworking employees who in return play their role to increase banks’ profitability (NIM). Opposite to our expectation, the coefficients of banking sector development show a significant negative relationship with all profitability indicators. It indicates banking sector development increased competition among banks which negatively affected the profitability of banks. This result is consistent with the findings of [124]. As our expectation, the economic growth of the country positively affects the banks’ profitability (NIM). On the other hand, opposite to our expectation infrastructure development which is measured by the number of mobile subscribers per 100 persons in the country negatively affected banks’ profitability (NIM). It’s because the cellular companies are offering many shadow banking services in Pakistan. These services include money transfers and holding cash in customers’ mobile accounts. So, by using these services people can send and receive money just showing their national identity cards. This practice is very common in every village and city in all over Pakistan. So, the customers prefer small money transactions through mobile companies rather than going to the banks because it is more convenient for them. So in this way, mobile companies are sharing banks’ profits which negatively affect banks’ interest margins. The results of dummy variables showed financial crises negatively affected the banks’ profitability (ROE and PBT). The Islamic banks showed higher profitability as compared to private banks in terms of ROA, ROE and PBT. The foreign banks also performed better than private banks in terms of ROA and PBT.

**Table 7:** When the competition is measured through the Lerner index.

	(1)		(2)		(3)		(4)	
	ROE		ROA		NIM		PBT/TA	
L.	0.354**	(3.64)	0.186**	(3.18)	0.671***	(11.97)	0.269***	(3.89)
Lerner index	0.138**	(3.19)	0.0205***	(5.40)	0.0149***	(6.51)	0.0293***	(4.27)
Financial crises	-0.0359***	(-4.14)	-0.00331**	(-2.87)	0.00180*	(2.24)	-0.00487***	(-4.09)
Size	0.0353***	(6.21)	0.00127*	(2.60)	0.000509	(1.00)	0.00193*	(2.28)
Capitalization	0.580***	(4.63)	0.0264*	(2.73)	0.0319***	(4.62)	0.0507***	(4.82)
Taxation	0.0415	(1.84)	0.00405*	(2.07)	-0.000697	(-0.28)	0.00717	(1.76)
Operational cost management	-0.946	(-1.64)	-0.0739*	(-2.63)	0.172*	(2.61)	-0.132**	(-2.99)
Credit risk	-0.441***	(-4.44)	-0.0111	(-0.82)	-0.0247	(-1.91)	-0.0210	(-0.96)
Liquidity risk	0.0533	(0.30)	-0.0208	(-0.90)	-0.0181	(-1.12)	-0.0403*	(-2.57)
Insolvency risk(Z-score)	0.0000806	(1.99)	0.0000114***	(3.78)	-0.000000934	(-0.28)	0.0000134*	(2.73)
Banking sector development	-0.469***	(-4.61)	-0.0442***	(-5.53)	-0.0559***	(-6.47)	-0.0527**	(-3.31)
GDP growth rate	0.0908	(0.27)	0.0161	(0.56)	0.113**	(3.35)	0.0201	(0.51)
Infrastructure development	0.000639	(0.70)	0.00000609	(0.07)	-0.000250**	(-3.21)	0.0000826	(0.99)
State owned banks	0.00155	(0.70)	-0.0156	(-1.98)	-0.000732	(-0.81)	-0.00171	(-1.26)
Islamic banks	0.00283	(2.04)	0.0513***	(4.20)	0.00190*	(2.41)	0.00258**	(2.81)
Foreign banks	-0.00286	(-1.19)	0.0569	(2.00)	0.00748**	(2.83)	0.0132***	(3.79)
Constant	-0.208*	(-2.09)	0.0109	(0.98)	0.0391**	(3.36)	0.000668	(0.04)
Observations	260		260		260		260	
No. of instruments	24		24		24		24	
F-test	174.8		108.5		566.9		241.6	
AR1	0.00489		0.00149		0.00224		0.00108	
AR2	0.564		0.575		0.835		0.574	
Hansen-J test	0.104		0.129		0.157		0.119	

*t* statistics in parentheses.

^*^ *p* < 0.05,

^**^ *p* < 0.01,

^***^ *p* < 0.001

where *,**,*** indicates that coefficients are significant at 5%,1% and 0.1% respectively.

Table 8 identifies that competition measured by the Boone indicator has a significant positive relationship with profitability (NIM, ROE and PBT) which indicates higher competition in the Pakistan banking industry led to decrease profitability. The significant positive coefficients of size for all profitability indicators showed that the profitability of the Pakistani banks increases with size. The significant positive coefficients of capitalization identify that well-capitalized banks are supposed to earn more profits which is consistent with the findings of [121, 125, 122]. The operational cost management negatively affected the profitability indicators (ROA and PBT). The credit risk negatively influenced the return on equity. The negative significant coefficient of liquidity risk indicates that holding more liquid assets negatively affect the banks’ profitability (NIM). The significant positive coefficients of Z-score indicate that more stable banks having less insolvency risk get more profits (ROE, ROA and PBT). Banking sector development increased the competition among Pakistani banks which negatively affected banks’ profitability (ROE, ROA and PBT). Islamic and foreign banks showed higher profitability (ROE, ROA and NIM) than private banks. The state-owned banks received more net interest margins and less return on equity as compared to private banks in Pakistan.

**Table 8:** When the competition is measured through the Boone indicator.

	(1)		(2)		(3)		(4)	
	ROE		ROA		NIM		PBT/TA	
L.	0.402***	(6.30)	0.208**	(3.18)	0.880***	(14.84)	0.362***	(4.47)
Boone indicator(ROA)	0.00145***	(4.24)	0.0000639	(1.76)	0.000128**	(3.35)	0.000192***	(5.84)
Financial crises	-0.0373	(-1.17)	-0.00310	(-1.02)	-0.00146	(-0.24)	-0.000246	(-0.05)
Size	0.0538***	(4.89)	0.00366***	(10.76)	0.00293***	(3.94)	0.00472***	(5.72)
Capitalization	0.643***	(4.36)	0.0394**	(3.37)	0.0729**	(3.39)	0.0605**	(3.60)
Taxation	0.110*	(2.79)	0.0100**	(3.28)	-0.00803	(-2.05)	0.0170**	(3.61)
Operational cost management	-0.755	(-1.59)	-0.112*	(-2.07)	0.0942	(1.78)	-0.118*	(-2.71)
Credit risk	-0.545***	(-5.91)	0.00345	(0.39)	-0.0347	(-1.91)	-0.00675	(-0.75)
Liquidity risk	0.245	(1.23)	0.00575	(0.28)	-0.104***	(-4.07)	0.0194	(0.66)
Insolvency risk (Z-score)	0.000200***	(5.94)	0.0000198***	(6.21)	0.00000332	(1.14)	0.0000238***	(3.97)
Banking sector development	-0.587**	(-3.54)	-0.0471***	(-4.58)	-0.0429	(-1.59)	-0.0888**	(-2.82)
GDP growth rate	-1.019	(-0.85)	0.0127	(0.13)	-0.0201	(-0.10)	0.167	(0.86)
Infrastructure development	0.00312	(1.94)	0.0000384	(0.37)	-0.000198	(-1.00)	-0.0000612	(-0.34)
State owned banks	0.00414**	(3.15)	-0.0260*	(-2.36)	0.000257	(1.71)	0.00587	(1.71)
Islamic banks	0.00265	(1.91)	0.0418***	(3.91)	0.00345***	(4.30)	0.00865**	(3.06)
Foreign banks	0.00161	(0.60)	0.0982***	(8.07)	0.0122***	(4.59)	0.0197*	(2.72)
Constant	-0.542*	(-2.23)	-0.0250*	(-2.51)	0.00720	(0.33)	-0.0194	(-0.94)
Observations	183		183		183		183	
No. of instruments	25		25		25		25	
F-test	2446.0		4489.8		3099.7		6102.3	
AR1	0.0195		0.0371		0.0467		0.0505	
AR2	0.207		0.894		0.929		0.136	
Hansen-J test	0.286		0.463		0.864		0.251	

*t* statistics in parentheses.

^*^ *p* < 0.05,

^**^ *p* < 0.01,

^***^ *p* < 0.001

where *,**,*** indicates that coefficients are significant at 5%,1% and 0.1% respectively.

### Robustness test

The robustness of the results is checked by the fixed-effect model. We choose between fixed effect and random effect models based on the Hausman test. The results show the positive coefficients of the Lerner index in Table 9 for all four models, which are consistent with our main model. However, when the competition is measured with the Boone indicator in Table 10, it showed a positive association with ROA and NIM but showed no significant relationship with ROE and PBT. Overall results of both robust models support the Structure-conduct-performance hypothesis which is consistent with the results of our main GMM model. The results of risk indicators (loan loss provisions and Z-score) also support our main estimation findings. The liquidity risk showed no significant relationship with profitability in the fixed-effect models.

**Table 9:** Fixed effect model when the competition is measured with the Lerner index.

	(1)		(2)		(3)		(4)	
	ROA		ROE		NIM		PBTTA	
Lerner	0.0195***	(7.45)	0.240***	(6.53)	0.0264***	(9.34)	0.0264***	(7.47)
Financial crises	-0.00431***	(-3.74)	-0.0526**	(-3.24)	0.00248*	(1.98)	-0.00667***	(-4.27)
Size	0.000665	(0.53)	0.0384*	(2.19)	0.00270*	(2.00)	0.000501	(0.30)
Capitalization	0.0277**	(2.95)	0.449***	(3.40)	0.0346***	(3.39)	0.0386**	(3.04)
Taxation	0.00272	(0.77)	0.0529	(1.06)	0.00111	(0.29)	0.00417	(0.87)
Operational cost management	-0.211***	(-3.80)	-1.163	(-1.49)	0.490***	(8.13)	-0.329***	(-4.36)
Credit risk	-0.0108	(-1.04)	-0.356*	(-2.44)	-0.0265*	(-2.37)	-0.0188	(-1.34)
Liquidity risk	-0.0176	(-1.16)	-0.176	(-0.82)	0.0183	(1.11)	-0.0283	(-1.37)
Insolvency risk (Z-score)	0.0000135***	(5.37)	0.000120***	(3.40)	0.00000113	(0.41)	0.0000188***	(5.53)
Banking sector development	-0.0375**	(-2.76)	-0.168	(-0.88)	-0.0768***	(-5.22)	-0.0406*	(-2.21)
GDP growth rate	0.0516	(1.52)	0.296	(0.62)	0.00752	(0.20)	0.0483	(1.05)
Infrastructure development	0.0000231	(0.38)	-0.000468	(-0.55)	-0.000173**	(-2.65)	0.000150	(1.84)
Constant	0.0137	(1.03)	-0.304	(-1.63)	0.0300*	(2.09)	0.0139	(0.77)
Observation	286		286		286		286	
Year FE	NO		NO		NO		NO	
F-test	22.42		16.29		25.65		25.91	
R-Squared	0.6736		0.6608		0.4691		0.6631	

*t* statistics in parentheses.

^*^ *p* < 0.05,

^**^ *p* < 0.01,

^***^ *p* < 0.001

where *,**,*** indicates that coefficients are significant at 5%,1% and 0.1% respectively.

**Table 10:** Fixed effect model when the competition is measured through the Boone indicator.

	(1)		(2)		(3)		(4)	
	ROA		ROE		NIM		PBTTA	
Boone indicator(ROA)	0.000218***	(5.21)	0.000977	(0.32)	0.0000970*	(2.89)	0.000404	(1.35)
Financial crises	-0.00306*	(-2.42)	-0.0375*	(-2.15)	0.00412**	(2.85)	-0.00493**	(-2.89)
Size	0.000890	(0.61)	0.0437*	(2.19)	0.00347*	(2.10)	0.000474	(0.24)
Capitalization	0.0306**	(2.90)	0.496***	(3.41)	0.0406***	(3.37)	0.0412**	(2.89)
Taxation	0.00129	(0.33)	0.0386	(0.71)	-0.000198	(-0.04)	0.00177	(0.33)
Operational cost management	-0.277***	(-4.53)	-1.942*	(-2.31)	0.407***	(5.84)	-0.421***	(-5.11)
Credit risk	-0.0290*	(-2.56)	-0.569***	(-3.65)	-0.0493***	(-3.81)	-0.0449**	(-2.93)
Liquidity risk	0.00456	(1.24)	0.0130	(0.26)	0.00196	(0.47)	0.00647	(1.30)
Insolvency risk (Z-score)	0.0000185***	(6.89)	0.000184***	(4.97)	0.00000829**	(2.70)	0.0000254***	(7.00)
Banking sector development	-0.0416**	(-2.77)	-0.219	(-1.06)	-0.0824***	(-4.82)	-0.0460*	(-2.27)
GDP growth rate	0.0588	(1.54)	0.428	(0.81)	0.0254	(0.58)	0.0522	(1.01)
Infrastructure development	0.0000414	(0.57)	-0.000405	(-0.40)	-0.000178*	(-2.14)	0.000213	(0.67)
Constant	0.0157	(1.02)	-0.305	(-1.44)	0.0280	(1.60)	0.0199	(0.96)
Observations	286		286		286		286	
Year FE	NO		NO		NO		NO	
F-test	13.21		12.98		18.88		17.73	
R-Squared	0.4978		0.6323		0.6821		0.5632	

*t* statistics in parentheses.

^*^ *p* < 0.05,

^**^ *p* < 0.01,

^***^ *p* < 0.001

where *,**,*** indicates that coefficients are significant at 5%,1% and 0.1% respectively.

## Conclusion

The objective of this study was to observe the impact of risk and competition on the profitability of Pakistani banking industry over the period of 2007–2017. The robustness of the results is checked by various risk and competition measures but being more specific we used the Lerner index and the Boone indicator techniques for measuring competition. The Z-score, Loan loss provisions and liquidity risk were used for capturing various types of risks in the Pakistani banking industry. We also controlled for comprehensive bank-specific, industry-specific and macroeconomic determinants of the banks’ profitability in addition to risk and competition. The two-step GMM model with the collapse command was used for econometric estimation. The empirical results show the significant positive value of the Lerner for all profitability indicators which identifies that competition negatively affects the profitability of Pakistani banks; it is in line with the Structure-Conduct-Performance (SCP) hypothesis. It identifies that the efficient structure hypothesis does not prevail yet in the Pakistani banks. The findings further show that risk measured by Z-score has a significant positive relationship with profitability (ROE, ROA and PBT), which identifies that lower risk leads to higher profitability because a higher value of the Z-score means lower risk. The credit risk also showed a negative impact on profitability (NIM).

Further results recognized the significant negative relationship between liquidity risk and profitability (ROE and NIM), which indicates that keeping the lower amount of liquid assets and a higher degree of loan exposure (lower liquidity) leads to more profitability. Besides, it was also observed that larger banks had more profitability with all profitability indicators because larger banks had better borrower screening and efficient risk portfolios. Further results also pointed out that Pakistani banks enjoyed more profitability during an economic boom. Finally, we found that the Islamic banks and foreign banks have higher ROA, PBT and NIM as compared to the private banks. The state-owned banks have higher NIM and lower ROE than private banks.

The findings of the study may help the policymakers, regulatory authorities and managers of Pakistani banks to take some policy measures to improve profitability.

❖ Some entry barriers should be introduced in the banking market to control competition, as the higher competition tends to take more risks and negatively affected banks' profitability in Pakistan.
❖ Banks should be encouraged to engage in more traditional loan activities because holding more liquid assets caused a decline in banks' profitability. This should be done with effective borrowers screening and efficient loan portfolios to mitigate loan defaults and increase profitability through interest income. Involving more lending activities will not only increase banks' profitability but also provide investment opportunities to entrepreneurs by providing timely loans.
❖ Banks should keep the focus on reducing their operational expenses but not at the cost of hiring competent employees because skillful workers help to increase banks' profits even though an increase in operational cost.
❖ Finally, the political influence in state-owned banks should be reduced to let them perform better.

This study had several limitations based on which future research could be focused on several directions. This study was limited to observe the impact of risk and competition on the profitability of banks by ignoring the other factors partaking in total productivity. So, future research could be conducted by examining the impact of risk and competition on the total factor productivity of banks. Future research could also include some other variables which are not included in this study, like banking regulations, exchange rate fluctuations, and mergers and acquisitions. Future studies could also use alternative methods of risk measurement like credit risk with a loan to deposit ratio, and liquidity risk with the volatility ratio of cash to total customer deposits from its trend.

## Supporting information

S1 Table Annual summary statistics for variables of Lerner index and Boone indicator. Note: w1 represents price of funds that is measured by the ratio of interest expense to total deposits. W2 indicates price of labor that is measured by personnel expanses to total assets ratio. W3 is price of capital which is equal to the Administrative and other operating expenses/ Total assets.
(PDF)

S2 Table When the competition is measured through the Lerner index. Note: This table exhibits two-step GMM regression output where Lerner index is used as competition measure; t-values are represented in parenthesis.
(PDF)

S3 Table When the competition is measured through the Boone indicator. Note: This table shows the results of the two-step GMM regression where the Boone indicator is used as competition measure, t-values are represented in parenthesis.
(PDF)
