# Supplementary material for: Impacts of risk and competition on the profitability of banks: Empirical evidence from Pakistan
Source: PLoS One. 2019 Nov 11;14(11):e0224378. doi: 10.1371/journal.pone.0224378 (PMC6844457; doi:10.1371/journal.pone.0224378)
Supplement: S1 Table — Note: w1 represents price of funds that is measured by the ratio of interest expense to total deposits. W2 indicates price of labor that is measured by personnel expanses to total assets ratio. W3 is price of capital which is equal to the Administrative and other operating expenses/ Total assets. (PDF) [file pone.0224378.s001.pdf]

Annual Summary Statistics for variables of Lerner Index and Boone indicator

Year	W1	W2	W3	Pi	MC	Lerner Index	Boone indicator
2007	.0364322	.01245713	.02581143	.08373516	.07279063	.09977323	.00851792
2008	04646462	.01518557	.0332607	.10073147	.0883709	.11449802	-.00853367
2009	.05294363	.01391052	.030584	.40335343	.09889298	.06943276	.00020088
2010	.05153544	.01393136	.03022524	.0999831	.09482656	.01983561	-.01593579
2011	.05437221	.0134352	.02910839	.10302626	.09648965	.05075253	.0008781
2012	.04962281	.01692195	.02810829	.09422275	.08926454	.0408826	.00087407
2013	.04294531	01278527	.02698121	.08465388	.08003723	.03805849	-.00520213
2014	.04293807	.01223587	.02602082	.09208543	.08041801	.09162116	-.00498306
2015	03472991	.01161805	.02331524	.07920019	.06936003	-.05530439	-.00613646
2016	.0292789	.01109036	.02333594	.06968437	.06154025	.08814668	-.0006358
2017	.02882734	.010524	.02330783	.06706224	.0617442	.07317504	-.00255245

STATA commands to measure Lerner index and Boone indicator

Do file commands for measuring Lerner index//

```
constraint define 1 lnw1+ lnw2 + lnw3 = 1

constraint define 2 lnqlnw1 + lnqlnw2 + lnqlnw3 = 0

constraint define 3 lnw12 + lnw1lnw2 + lnw1lnw3 = 0

constraint define 4 lnw22 + lnw1lnw2 + lnw2lnw3 = 0

constraint define 5 lnw32 + lnw1lnw3 + lnw2lnw3 = 0

cnsreg lnc lnq lnq2 lnw1 lnw2 lnw3  lnw12 lnw22 lnw32 lnqlnw1 lnqlnw2 lnqlnw3 lnw1lnw2
lnw1lnw3 lnw2lnw3, constraints(1 2 3 4 5)

gen seq = _b[lnq] + _b[lnq2]*lnq + _b[lnqlnw1]*lnw1 + _b[lnqlnw2]*lnw2 + _b[lnqlnw3]*lnw3

gen mc = seq * (c/q)

gen lerner = (pi - mc)/pi

sum mc lerner pi

collapse ( mean ) lerner mc pi , by( t )//
```

Command used to measure Boone indicator//

```
xtset id t

bys t: asreg ROA lnMC , wind( t 11)//
```
