# Supplementary material for: Impacts of risk and competition on the profitability of banks: Empirical evidence from Pakistan
Source: PLoS One. 2019 Nov 11;14(11):e0224378. doi: 10.1371/journal.pone.0224378 (PMC6844457; doi:10.1371/journal.pone.0224378)
Supplement: S2 Table — Note: This table exhibits two-step GMM regression output where Lerner index is used as competition measure; t-values are represented in parenthesis. (PDF) [file pone.0224378.s002.pdf]

	(1) ROE		(2) ROA		(3) NIM		(4) PBT/TA	
L.	0.354**	(3.64)	0.186**	(3.18)	0.671***	(11.97)	0.269***	(3.89)
Lerner index	0.138**	(3.19)	0.0205***	(5.40)	0.0149***	(6.51)	0.0293***	(4.27)
Financial crises	-0.0359***	(-4.14)	-0.00331**	(-2.87)	0.00180*	(2.24)	-0.00487***	(-4.09)
Size	0.0353***	(6.21)	0.00127*	(2.60)	0.000509	(1.00)	0.00193*	(2.28)
Capitalization	0.580***	(4.63)	0.0264*	(2.73)	0.0319***	(4.62)	0.0507***	(4.82)
Taxation	0.0415	(1.84)	0.00405*	(2.07)	-0.000697	(-0.28)	0.00717	(1.76)
Operational cost management	-0.946	(-1.64)	-0.0739*	(-2.63)	0.172*	(2.61)	-0.132**	(-2.99)
Credit risk	-0.441***	(-4.44)	-0.0111	(-0.82)	-0.0247	(-1.91)	-0.0210	(-0.96)
Liquidity risk	0.0533	(0.30)	-0.0208	(-0.90)	-0.0181	(-1.12)	-0.0403*	(-2.57)
Insolvency risk(Z-score)	0.0000806	(1.99)	0.0000114***	(3.78)	-0.000000934	(-0.28)	0.0000134*	(2.73)
Banking sector development	-0.469***	(-4.61)	-0.0442***	(-5.53)	-0.0559***	(-6.47)	-0.0527**	(-3.31)
GDP growth rate	0.0908	(0.27)	0.0161	(0.56)	0.113**	(3.35)	0.0201	(0.51)
Infrastructure development	0.000639	(0.70)	0.00000609	(0.07)	-0.000250**	(-3.21)	0.0000826	(0.99)
State owned banks	0.00155	(0.70)	-0.0156	(-1.98)	-0.000732	(-0.81)	-0.00171	(-1.26)
Islamic banks	0.00283	(2.04)	0.0513***	(4.20)	0.00190*	(2.41)	0.00258**	(2.81)
Foreign banks	-0.00286	(-1.19)	0.0569	(2.00)	0.00748**	(2.83)	0.0132***	(3.79)
Constant	-0.208*	(-2.09)	0.0109	(0.98)	0.0391**	(3.36)	0.000668	(0.04)
Observations	260		260		260		260	
No. of instruments	24		24		24		24	
F-test	174.8		108.5		566.9		241.6	
AR1	0.00489		0.00149		0.00224		0.00108	
AR2	0.564		0.575		0.835		0.574	
Hansen-J test	0.104		0.129		0.157		0.119	
