# Supplementary material for: Impacts of risk and competition on the profitability of banks: Empirical evidence from Pakistan
Source: PLoS One. 2019 Nov 11;14(11):e0224378. doi: 10.1371/journal.pone.0224378 (PMC6844457; doi:10.1371/journal.pone.0224378)
Supplement: S3 Table — Note: This table shows the results of the two-step GMM regression where the Boone indicator is used as competition measure, t-values are represented in parenthesis. (PDF) [file pone.0224378.s003.pdf]

	(1)<br>ROE		(2)<br>ROA		(3)<br>NIM		(4)<br>PBT/TA	
L.	0.402***	(6.30)	0.208**	(3.18)	0.880***	(14.84)	0.362***	(4.47)
Boone indicator(ROA)	0.00145***	(4.24)	0.0000639	(1.76)	0.000128**	(3.35)	0.000192***	(5.84)
Financial crises	-0.0373	(-1.17)	-0.00310	(-1.02)	-0.00146	(-0.24)	-0.000246	(-0.05)
Size	0.0538***	(4.89)	0.00366***	(10.76)	0.00293***	(3.94)	0.00472***	(5.72)
Capitalization	0.643***	(4.36)	0.0394**	(3.37)	0.0729**	(3.39)	0.0605**	(3.60)
Taxation	0.110*	(2.79)	0.0100**	(3.28)	-0.00803	(-2.05)	0.0170**	(3.61)
Operational cost management	-0.755	(-1.59)	-0.112*	(-2.07)	0.0942	(1.78)	-0.118*	(-2.71)
Credit risk	-0.545***	(-5.91)	0.00345	(0.39)	-0.0347	(-1.91)	-0.00675	(-0.75)
Liquidity risk	0.245	(1.23)	0.00575	(0.28)	-0.104***	(-4.07)	0.0194	(0.66)
Insolvency risk(Z-score)	0.000200***	(5.94)	0.0000198***	(6.21)	0.00000332	(1.14)	0.0000238***	(3.97)
Banking sector development	-0.587**	(-3.54)	-0.0471***	(-4.58)	-0.0429	(-1.59)	-0.0888**	(-2.82)
GDP growth rate	-1.019	(-0.85)	0.0127	(0.13)	-0.0201	(-0.10)	0.167	(0.86)
Infrastructure development	0.00312	(1.94)	0.0000384	(0.37)	-0.000198	(-1.00)	-0.0000612	(-0.34)
State owned banks	0.00414**	(3.15)	-0.0260*	(-2.36)	0.000257	(1.71)	0.00587	(1.71)
Islamic banks	0.00265	(1.91)	0.0418***	(3.91)	0.00345***	(4.30)	0.00865**	(3.06)
Foreign banks	0.00161	(0.60)	0.0982***	(8.07)	0.0122***	(4.59)	0.0197*	(2.72)
Constant	-0.542*	(-2.23)	-0.0250*	(-2.51)	0.00720	(0.33)	-0.0194	(-0.94)
Observations	183		183		183		183	
No. of instruments	25		25		25		25	
F-test	2446.0		4489.8		3099.7		6102.3	
AR1	0.0195		0.0371		0.0467		0.0505	
AR2	0.207		0.894		0.929		0.136	
Hansen-J test	0.286		0.463		0.864		0.251	
